# Medical Students’ Online Learning Perceptions, Online Learning Readiness, and Learning Outcomes during COVID-19: The Moderating Role of Teacher’s Readiness to Teach Online

**DOI:** 10.3390/ijerph19063520

**Published:** 2022-03-16

**Authors:** Muddassar Sarfraz, Ghulam Hussain, Muhammad Shahid, Amir Riaz, Muhammad Muavia, Yahya Saleem Fahed, Faiza Azam, Mohammad Tallal Abdullah

**Affiliations:** 1 College of International Students, Wuxi University, Wuxi 214105, China; muddassar.sarfraz@gmail.com; 2 Department of Management Sciences, COMSATS University Islamabad, Lahore Campus, Lahore 54000, Pakistan; amirriaz@cuilahore.edu.pk (A.R.); muhammad.muavia@live.com (M.M.); 3 Postgraduate Medical Institute, Ameer-ud-Din Medical College, Lahore General Hospital, Lahore 54000, Pakistan; drshahid007@hotmail.com; 4 Department of Pathology, Government Said Mitha Teaching Hospital, Lahore 54000, Pakistan; drysf12@gmail.com; 5 Department of Pathology, Akhtar Saeed Medical and Dental College, Lahore 54000, Pakistan; fiz60azam@yahoo.com; 6 Department of Orthopaedic Surgery, Continental Medical College, Lahore 54000, Pakistan; tallalabdullah@hotmail.com

**Keywords:** students’ online learning perceptions (SOLPs), students’ readiness for online learning (SRFOL), learning outcomes (LO), teachers’ online teaching readiness (TOTR), medical education

## Abstract

This study determined the direct and indirect effects of medical students’ online learning perceptions on learning outcomes via their readiness for online learning. It also determined the moderating effect of teachers’ online teaching readiness on medical students’ online learning perceptions and learning outcomes. We apply the theoretical lens of self-determination theory and constructivist theory to formulate hypotheses. We used self-administered and postal survey methods to collect data from fourth and fifth-year medical students on online learning perceptions, readiness for online learning, and learning outcomes in two waves. We also collected data from the teachers about their perceptions of online teaching readiness. We received 517 usable students’ responses (Level-1) and 88 usable teachers’ responses (Level-2). We tested Level-1 hypotheses about direct and indirect effects in Analysis of Moment Structures (AMOS), and a Level-2 hypothesis about moderating effect was tested using Hierarchical Linear Modeling (HLM). The results for the Level-1 hypotheses supported the positive effects of students’ online learning perceptions and readiness for online learning on learning outcomes. Student readiness for online learning significantly mediated the relationship between online learning perceptions and learning outcomes. HLM results also supported a moderating effect of teachers’ online teaching readiness on medical students’ online learning perceptions and learning outcomes in such a way that learning outcomes were high when students’ online learning perceptions and teachers’ online teaching readiness were high. Based on the study’s findings, we offer contributions to theory and practice.

## 1. Introduction

The outbreak of the COVID-19 pandemic has significantly affected every walk of life, and education in particular. As a result, educational institutions around the globe have shifted conventional teaching to online teaching [1]. The emergency shift from face-to-face education to online education has left unprecedented effects on teachers and students, requiring the immediate attention of researchers and policymakers to introduce and implement an online learning system that enables students to gain essential knowledge and skills [2,3,4]. This significant change has posed challenges to medical education that focuses both on knowledge (theory) and skills development (practical orientation) [1]. Especially for medical students, their direct patient-interaction and physical examination skills have been affected, raising serious concerns about inadequate preparation of the students who are about to begin their professional careers [5,6,7]. There have been recent calls for research to assess the effectiveness of online learning [8,9,10], particularly for the medical discipline [4,5,11]. Our study responds to such calls to assess the medical students’ online learning perceptions (SOLPs), their readiness for online learning, and learning outcomes (LO), considering the teachers’ online teaching readiness (TOTR) as a contingency factor.

Online learning encompasses two factors: (a) “learning, a cognitive process to achieve knowledge, and (b) technology, an enabler of the learning process” [12]. Research has shown that SOLPs influence LO and performance in an online learning environment [13,14]. Students’ attitude towards online learning determines their acceptance level of online learning, achievement of LO, and future use of online learning platforms [8,9,15]. The link between online learning perceptions and LO entails the intervening mechanisms that help to explain this relationship [13].

The literature suggests students’ readiness for online learning (SRFOL) works as an intervening mechanism between online learning perceptions and LO [9,13] that is built upon the premise of self-determination theory [16]. This theory states that individuals take volitional actions based on their own will that lead to the development of competencies. The developmental tendencies do not operate in isolation but require an environment to support them. Therefore, self-determined behaviors come from conscious choice. This theory also posits that “the choice of experience” is intrinsically motivated, but to some extent also present in the extrinsically motivated behaviors. Intrinsic motivation refers to autonomous motivation to undertake tasks for seeking satisfaction and pleasure. For example, the students engage in online learning who enjoy learning and are interested in subjects. Extrinsic motivation refers to undertaking tasks because of instrumental reasons. For example, students are motivated to pass an exam to earn grades, appreciation, and/or avoid punishment [16]. Online learning provides flexible, self-paced, customized learning and better opportunities for the students to interact with peers, instructors, and specialized groups that maximize the learning quality [9,13]. Lou [17] asseverated that the students in an online learning environment are intrinsically motivated to share their knowledge with their fellows. Therefore, they become ready to adopt online learning to achieve LO. Thus, this study aims to determine the direct and indirect effects of SOLPs on LO via their readiness to learn online [8,9].

Deci and Ryan [18] asseverated environmental forces could support or hinder self-determination. A teacher’s role is significant in influencing the students’ self-determination and its consequences in an educational context. Besides self-determination theory, the constructivism theory provides an important theoretical lens for understanding the students’ online learning experiences through social interactions with the teachers [19]. Therefore, TOTR is imperative in implementing online learning and to help the students’ achievement of LO [11,14,20,21]. The success of an online learning system also depends on the teachers’ competencies and skills to adapt to the online learning environment [2,10]. Thus, this study aims to determine the moderating effect of TOTR on SOLPs and LO.

This research makes significant contributions to theory and practice. First, we offer novel insights by highlighting the relationship between medical SOLPs and LO via their readiness to learn online. We establish the direct and indirect relationships upon the premise of self-determination theory [16]. This theory has been widely used in many fields, such as workplace, sports, psychotherapy, and education (primary, secondary, and tertiary education). However, to the best of our knowledge, the application of self-determination theory, especially in medication education, is scant. Therefore, this study attempts to apply and test the theoretical lens of the self-determination theory in medical education. Second, our study extends self-determination theory and social constructivism theory by proposing the TOTR as a moderator of SOLPs and LO. Upon the premise of social constructivism, we posit that a lesson that is designed and implemented to offer opportunities to learn could be labeled as a constructivist lesson that leads to the achievement of LO [1,19]. This will help to determine the extent to which intrinsic and extrinsic motivations are critical to the success of online learning [17].

Third, we selected a sample of fourth- and fifth-year medical students enrolled in an online course. Earlier studies in this domain had selected students’ samples other than the medical field. However, medical education presents a unique case for investigating the underlying phenomena because of its focus on preparing a doctor who should be educated both in theory and practice [22]. In particular, the fourth- and fifth-year medical students are ready to embark on clinical practice. Research has shown that practical orientation is not only identified in teaching content but also in teaching pedagogy [2,5,22]. Because of the COVID-19 outbreak, face-to-face classroom teaching is switched to online teaching to ensure the supply of skilled healthcare professionals to cater to the healthcare system’s needs. Thus, it has posed a monumental challenge to medical education whether the medical students can gain the desired level of knowledge and skills in online learning [5]. Fourth, earlier researchers established the links between online learning perceptions and course satisfaction and performance [13]. However, researchers in the medical field emphasized that measuring course LO in terms of knowledge and skills acquisitions is more important than course satisfaction and course performance in terms of grade [4,11]. The practical knowledge and skills gained by the medical students are more important for their clinical practice than their course satisfaction and grade. Because of the COVID-19 outbreak, formal assessments conducted by the teachers are compromised [21]. Therefore, we included self-rated LO as a criterion variable in our study.

The rest of this manuscript comprises four sections. First, we formulate hypotheses with the help of classical and contemporary literature. Second, we discuss the research methodology employed to test the hypotheses. Third, we present the results of the study. Last, we discuss the study’s results and present theoretical and practical implications.

## 2. Theoretical Background and Hypotheses Development

### 2.1. Online Learning Perceptions and Learning Outcomes

The growing use of internet technologies in education has resulted in new learning paradigms, such as online learning [23]. Over time, different terminologies such as online learning, e-learning, distributed learning, virtual learning, computer-assisted learning, distance learning, and web-based learning have been presented, making it difficult to propose a single definition for online learning [13]. The focus of the scholars remains on proposing an online learning process that is positively viewed by the learners so that they can achieve LO [10]. Online learning perceptions refer to learners’ attitudes towards computer and technology-oriented education [15].

Wei and Chou [13] synthesized and redefined the learner’s online learning perceptions in response to fragmentation in online learning literature. Their definition encompasses five dimensions of online learning perceptions that include accessibility, interactivity, adaptability, knowledge acquisition, and ease of loading. Accessibility refers to the availability and free access to course materials and other learning resources [13]. Interactivity refers to sociability and considers learning an interactive knowledge acquisition process through online discussion with peers, classmates, and instructors, such as asking questions and discussing issues [13,24]. Adaptability refers to the learner’s ability to control the learning process, such as deciding when and where to learn. They defined knowledge acquisition as a learner’s ability to gain new knowledge that he/she seeks to broaden his/her horizon. Ease of loading refers to learners’ perceptions about the lower burden and less stress in an online learning environment [13].

The learner’s characteristics are critical in that they affect the success of online learning. Therefore, a learner-positive attitude towards online learning contributes to success [8,25]. Because of the COVID-19 outbreak, online learning is a way to continue learning [26]. The research shows that learners’ positive perceptions of online learning enable them to perceive more support and benefits in online learning that enhance their learning [13]. They perceive online learning as an effective mode of education that offers access to course material, efficient time management, cost-effectiveness, flexibility to learn from anywhere, and opportunities to collaborate and work with peers and instructors to achieve LO [1,27,28]. Thus, the benefits of online learning develop students’ positive perceptions of online learning that enable him/her to achieve LO [8]. Therefore, we propose:

**H1**: *SOLPs positively relate to LO*.

### 2.2. Students’ Readiness for Online Learning as a Mediator

Learners’ readiness for online learning was first proposed by Warner [29]. McVay [30] refined it and proposed two dimensions of readiness for online learning that include “comfort with e-learning” and “self-management of learning.” Later, Hung [27] broadened the conceptual domain of learner’s readiness for online learning and presented its five dimensions: self-directed learning, motivation for learning, learner’s control, computer & internet self-efficacy, and online communication self-efficacy.

The concept of self-directed learning is based on the self-direct learner’s characteristics presented by Knowles [31]. It is defined as a process in which a learner assesses his/her learning needs, establishes LO, searches for the learning material and resources, employs the right learning strategies, and evaluates the LO. In a self-directed learning process, a learner takes responsibility for learning and shows enthusiasm about learning [27]. Motivation for learning is built upon the premise of both intrinsic and extrinsic motivational aspects [16]. Intrinsic motivation in the online learning context shows the learner’s interest in gaining new knowledge and skills to grow in his/her field. The learner becomes extrinsically motivated through good grades, awards, and prizes. The learner’s motivation for learning directs his/her efforts towards his/her learning desires, rehearsal, retention, and retrieval [27]. A learner’s control shows the degree to which a learner can direct his/her learning experiences and process [9]. The last two dimensions, “computer & internet self-efficacy” and “online communication self-efficacy,” are derived from social cognitive theory and the principle of general self-efficacy that refers to an individual’s belief about his/her abilities to undertake an activity [32]. The general self-efficacy does not capture and explain task-specific efficacy [27]. In an online learning environment, courses are delivered through a computer-mediated network. Therefore, computer & internet self-efficacy and online communication self-efficacy as task-specific efficacies explain a learner’s ability to undertake activities related to online learning. Internet & computer self-efficacy refers to the learner’s belief about his/her abilities to use the internet and computer in an online learning environment. Online communication self-efficacy refers to the learner’s judgment about his/her ability in using online tools to communicate effectively [27].

Michotte [33] argued that perception plays an important role in shaping the actions to adjust to the world where we live. The perception depicts a motivational force that influences learners’ motivational state towards online learning [16] and facilitates them to develop self-efficacy, positive emotions and undertake activities to accomplish outcomes [34]. Online learning during COVID-19 contributes to the intrinsic and extrinsic motivation of the students in such a manner that students view the online learning system as a way to gain knowledge and skills and complete their studies on time to embark on a professional career. Therefore, students’ positive perceptions of online learning foster their readiness to gain and develop essential competencies to adopt online learning [9]. Literature has also showed that readiness for online learning is associated with LO and course satisfaction [13,35]. The students who possess technology-related knowledge and skills, and have high confidence, are more likely to engage in online learning for achieving outcomes [9]. A recent study reported that SRFOL mediated the relationship between online learning perceptions and course performance and satisfaction [13]. Thus, in the light of the above discussion, we expect that:

**H2**: *SOLPs positively relate to their readiness for online learning*.

**H3**: *SRFOL positively relates to LO*.

**H4**: *SRFOL mediates the positive relationship between SOLPs and LO*.

### 2.3. Teachers’ Online Teaching Readiness

The assertions that (a) environmental forces support or hinder a learner’s self-determination [18], (b) construction of knowledge through social interaction with the teacher, and (c) incorporating the combination of intrinsic and extrinsic motivations for determining the effectiveness of online learning process need to explain the contingent effects of TOTR on the relationship between SOLPs and LO [23]. Martin [36] defined TOTR as their’ preparedness to teach online courses and presented four dimensions; course design, course communication, time management, and technical competencies specific to technology.

Course design is defined as a main pedagogical competency of the teacher that relates to defining course learning objectives and outcomes, selecting course materials and instructional strategies, and designing and administering the assessments that align with learning objectives and outcomes [36]. Course communication refers to the teacher’s ability to effectively communicate with students in a computer-mediated environment, such as passing instructions through web-based forums, chats, and emails about feedback delivery, rules and regulations, netiquettes, deadlines, course expectations, and ethical practices [36]. Time management refers to the teacher’s effective time-management skills [37]. Designing an online course for the first time takes a longer time as an instructor has to realign the entire course-related tasks according to the online format [36]. A teacher also spends more time helping the struggling students in addressing their queries and technical difficulties [2]. Technical competence specific to technology usage shows a teacher’s competence in using a learning management system, software for synchronous and asynchronous modes, ability to troubleshoot the technical issues and help students to use technology [36].

Teachers’ readiness for online teaching depicts two psychological states: attitude and ability. An attitude refers to the teacher’s belief of accepting that the online learning environment differs from face-to-face teaching, and ability refers to their competence to teach courses online [36]. The educational system’s success depends on teachers’ attitudes and beliefs [2]. Therefore, teachers who develop competencies for online teaching encourage their students to adopt online learning. A teacher facilitates the students to build and gain knowledge, develop essential skills, and solve problems [2].

The teachers’ skills and beliefs play an important role in defining and improving the success of technological-based learning [2]. Therefore, TOTR, in combination with SOLPs, enhances the achievement of LO in terms of knowledge and skills acquisition. This relationship is in line with self-determination theory, which posits that intrinsic and extrinsic motivations inspire the learners to the “choice of experience” that leads to competency development [18]. The congruence between SOLPs and TOTR positively contributes to the students’ intended actions for achieving LO [23]. Besides, self-determination theoretical lens, an immediate social context as instructor’s supportive behaviors, facilitates the learners to build and gain the desired knowledge and skills [27,36]. Therefore, we expect that:

**H5**: *TOTR moderates the relationship between SOLPs and LO*.

**Figure 1:**
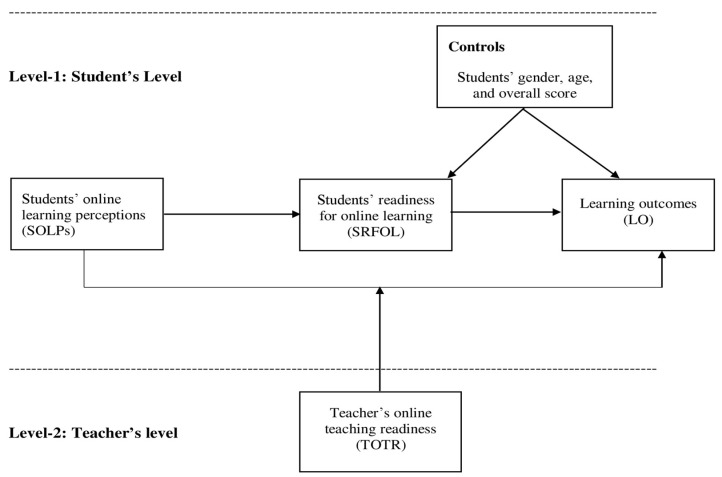
Conceptual framework.

The Figure 1 shows the conceptual framework of the study.

## 3. Methodology

### 3.1. Participants

The medical colleges offer medical education in Pakistan regulated by the Pakistan Medical Commission and provincial health departments. There are 176 medical and dental colleges in Pakistan that mainly offer undergraduate degrees such as Bachelor of Medicine, Bachelor of Surgery (MBBS), and Bachelor of Dental Surgery (BDS). Some colleges also offer specialized graduate programs. This study targeted the fourth- and fifth-year MBBS students who were enrolled in online courses and the faculty members who were teaching online courses in the medical colleges of the Punjab Province. There are 63 medical colleges in the Punjab Province that make up 35% of the total medical colleges in the country [38,39]. We collected data through self-administered and postal surveys. In designing the surveys, data collection, and reporting, we followed the ethical guidelines of our institutions. Each survey accompanied a cover letter that explained the study’s aim and assurance of data confidentiality to the respondents. Participation in the survey was voluntary, and the respondents had full rights to withdraw at any stage of the study, giving no reason.

We constructed three versions of the surveys: two for students (the first version comprised measures of SOLPs and SRFOL, and the second version that included the measure of LO) and the third for the faculty members to measure TOTR. In data collection from students, we employed a time-lagged design. First, the chosen design enabled keeping the cause-and-effect relationship’s temporal order [40]. Second, the dependent variable in our study was LO which could be better measured after the delivery of enough no. of online sessions. Third, it helps to overcome common method bias. Thus, we maintained a two-month lag in time between the first (T1) and second (T2) waves of data collection. We created a unique code for each respondent and class. We assigned the same code to the course instructor and students for response matching processes at a later stage. Each survey was accompanied by a cover letter. We briefly explained the purpose of our study and the anonymity of the responses.

### 3.2. Data Collection

Because of COVID-19, the most challenging task was to locate our study’s respondents. The authors mainly used their professional network to identify and locate the study’s respondents. We mainly employed the snow-ball sampling technique to maximize the response rate. This is not a random sample, but it is still of interest to apply statistical significance tests in this situation. At T1, we distributed over 1500 questionnaires to the students and over 200 questionnaires to the faculty members. We received 723 filled questionnaires from the students and 119 filled responses from the faculty members. Some questionnaires were incomplete or inappropriately filled. Therefore, we discarded all such responses, leaving 658 students’ responses and 103 faculty members’ responses. At T2, we administered 658 surveys to the students and retrieved 596 filled responses. We started a matching process by matching codes to identify the responses belonging to the same respondents and classes. Following earlier researchers’ guidelines on multi-level design, we employed four to eight students’ responses from a single course as a criterion for considering the valid response [41]. The matching process reduced the sample to 517 students and 88 faculty members.

The demographic results for the students’ sample show that 278 were male students and 239 were female students. Among 517 students, 317 students (166 males and 151 females) were in their fourth year, and 200 students (112 males and 88 females) were in the fifth year of their studies. The results further show that the average age of the students’ sample remained 25.42 years, with 1.97 years standard deviation.

The demographic analysis for the teachers’ sample shows that 52 respondents were male respondents and 36 were female respondents. We found the average age of the faculty members was 49.88 years, with 6.33 years standard deviation. Most of the faculty members (83) held a master’s degree, only five faculty members were Ph.D. degree holders. Their responses further show that 54 faculty members used Zoom, 26 faculty members used MS Teams, and eight used Google meet for online teaching.

### 3.3. Measurement Scales

#### 3.3.1. Students’ Online Learning Perceptions

We used a 23-item scale to measure SOLPs [13]. Of the 23 items: four items were for accessibility, adaptability, and ease of loading; six items for interactivity, and five were used for knowledge acquisition. The sample items include “online learning provides various online resources,” “online learning enables me to interact directly with other learners,” “online learning enables me to decide on the best time to learn,” “online learning enables me to learn more about the knowledge that I desire to learn” and “online learning environments can effectively reduce learning burden”.

#### 3.3.2. Students’ Readiness for Online Learning

We used a seventeen-item scale to measure the SRFOL [27]. Of the seventeen items: three items each were used for learner’s control, computer & internet self-efficacy, and online communication self-efficacy; and five items were for self-directed learning and four items for motivation for online learning. The sample items include “I feel confident in my knowledge and skills of how to manage software for online learning, “I carry out my own study plan, “I can direct my own learning progress, “I am open to new ideas” and “I feel confident in using online tools (email, discussion) to communicate with others effectively”.

#### 3.3.3. Learning Outcomes

There were a variety of scales to measure LO in the literature. Using an appropriate scale to measure LO for medical students was a major consideration. We constructed the scale to measure LO in various stages. First, we conducted a literature search and collected different measures of LO. The scrutiny of measures showed that the ‘Conceptions of Learning Medicine Questionnaire’ developed by Chiu [42] was specific to medical education. Second, we arranged focus group discussions and briefed the group about the study’s objectives and the purpose of the focus group discussion. We presented the ‘Conceptions of Learning Medicine Questionnaire’ for deliberation. They used the Q-sorted method and, after detailed deliberation, they finalized six items to measure LO. They recommended conducting a pilot study and to meet again if ambiguity arises. The respondents did not report any issue in a pilot study. Thus, the surveys were prepared to administer the larger groups for the main study.

#### 3.3.4. Teachers’ Online Teaching Readiness

We employed a thirty-one-item scale to measure TOTR [36]. Of the total, eight items for course design, ten items for course communication, six items for time management, and seven items for technical competence of the teachers in using technology for online teaching were used. Sample items include “design learning activities that offer students opportunities for interaction (e.g., discussion forums, wikis), “create and moderate discussion forums, “use features in learning management system in order to manage time (e.g., online grading, rubrics, SpeedGrader, calendar), and “navigate within the course in the learning management system (e.g., Moodle, Canvas, and Blackboard). Table A1 presents all the variables measurement scale items. 

### 3.4. Data Analysis Strategy

This study determined the direct and indirect effects of SOLPs on LO via SRFOL. It also determined the moderating role of TOTR on SOLPs and LO. The data were collected at two levels; SOLPs, SRFOL, and LO were conceptualized at individual student level of analysis (Level-1), whereas TOTR was conceptualized as a class-level variable (Level-2). Before testing the study’s hypotheses, the goodness of the scales was tested through confirmatory factor analysis in AMOS. We constructed separate measurement models for Level-1 and Level-2 variables. Besides, variables such as SOLPs, SRFOL, and TOTR are second-order scales that require testing the first-order and second-order measurement models separately. Therefore, four different measurement models were constructed and tested (See Section 4.1 for more detail).

This study tested Level-1 and Level-2 hypotheses. The level-1 hypotheses are mainly about the direct (H1–H3) and indirect effects (H4), and these were tested in AMOS by constructing a structural model. As for the multi-level hypothesis (H5), where students’ level variables (SOLPs, SRFOL, and LO) are nested within the class level variable (TOTR), hierarchical linear modeling (HLM) was used. It is a most appropriate and robust approach, having clear advantages over conventional regression approaches to test multilevel relationships [43].

## 4. Results

### 4.1. Measurement Models

#### 4.1.1. Confirmatory Factor Analysis for Level-1

**Figure 2:**
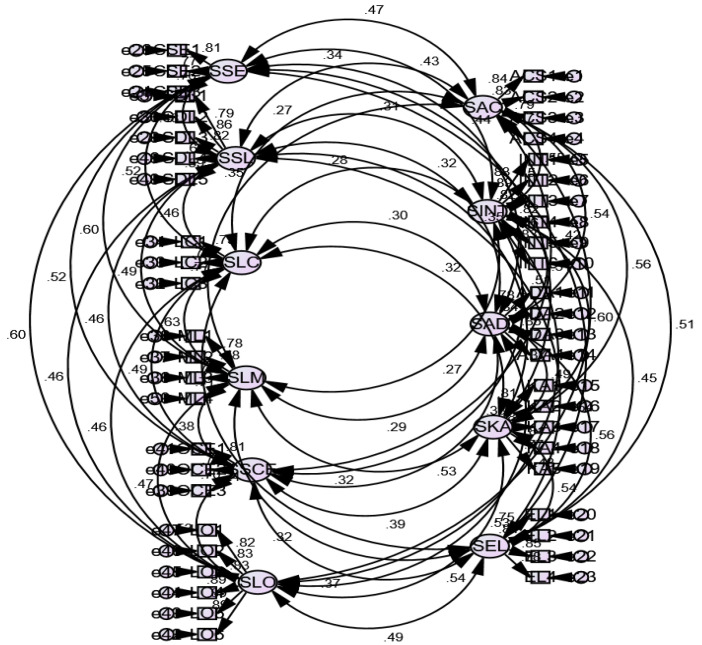
First order measurement model for student-level constructs (Eleven-factor measurement model).

A confirmatory factor analysis was employed to assess the validity of the measures used in this study. First, for student-level variables (Level-1), a first-order measurement model was specified in which all the indicators of the latent constructs were loaded on their respective constructs (Figure 2). The results (omitted due to space constraint) show that the loading scores of all indicators on their respective constructs are greater than 0.50 and significant at the level *p* < 0.01 [44]. We tested the convergent validity by computing the average variance extracted (AVE) scores for each variable. The results showed AVE scores in all cases exceeded 0.50, which supported the convergent validity [44]. We tested discriminant validity by comparing the squared roots of AVE with paired correlation coefficients. This comparison showed that the squared roots of AVE scores are greater than paired correlation that confirmed discriminant validity [43]. 

**Figure 3:**
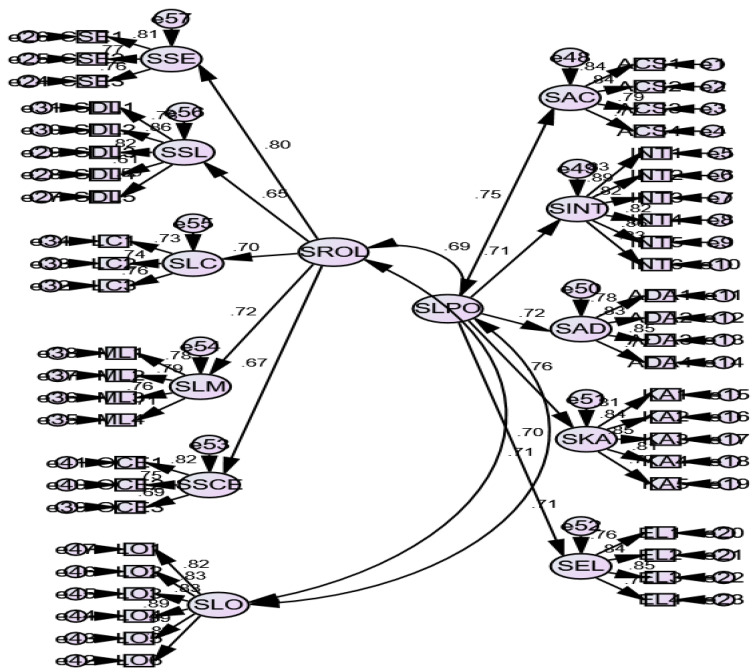
Second-order measurement model for student-level constructs (Level-1: three-factor model).

Next, we specified a second-order measurement model comprising three factors: SOLPs, SRFOL (second-order constructs), and LO (Figure 3).

The results presented in Table 1 showed that factor loading scores in the second-order measurement model are also greater than 0.50 and significant at the level *p* < 0.01. We computed AVE scores by using standardized loading scores. The results showed that AVE scores were greater than 0.50, which supported the convergent validity of second-order latent constructs. We tested discriminant validity by employing Fornell–Larcker’s criterion [45]. The fit indices of both student-level measurement models (eleven-factor model and three-factor model) presented in Table 2 showed better fit. A three-factor second-order measurement model showed a better fit (Table 2: CMIN/DF = 1.98, RMR = 0.03, RMSEA = 0.04, IFI = 0.94, CFI = 0.94, TLI = 0.94).

Besides validity, we tested reliability by obtaining the values of Cronbach’s Alpha (α) and composite reliability. The results showed that in all cases, Cronbach’s Alpha values (α) and composite reliability were greater than 0.70 and 0.80, respectively, supporting the reliability [46].

#### 4.1.2. Confirmatory Factor Analysis for Level-2

**Figure 4:**
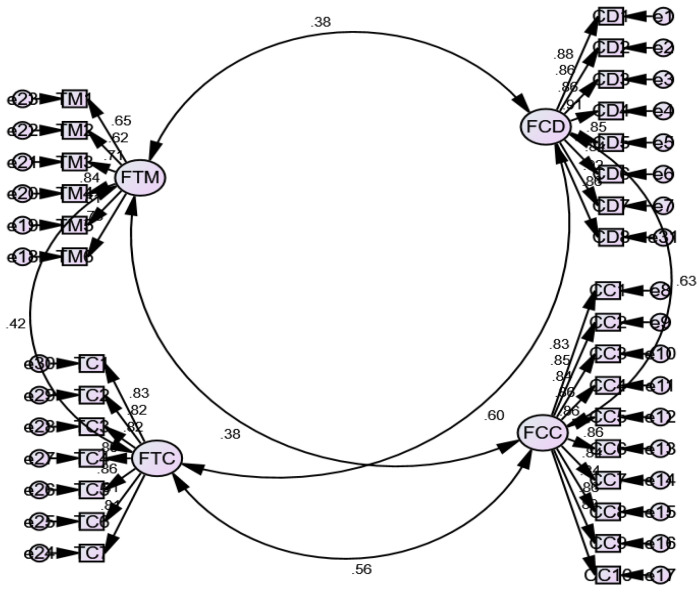
First-order measurement model for teacher-level constructs (four-factor measurement model).

**Figure 5:**
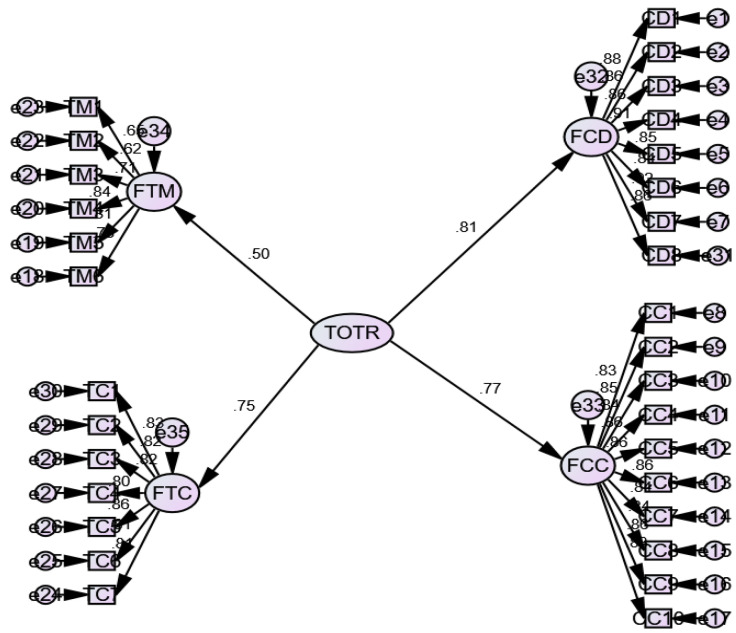
Second-order measurement model for teacher-level constructs.

Further, for the teacher-level construct (Level-2), first, we conducted a first-order measurement model (Figure 4). Second, we conducted a second-order measurement model (Figure 5).

The results met the criteria for convergent (Table 3) and discriminant validities (Table 4). Both measurement models had better fit indices (Table 2). The values of Cronbach’s Alpha (α) and composite reliability for the first and second-order constructs were also greater than 0.70 and 0.80, respectively, showing good reliability [46].

### 4.2. Hypotheses Testing

#### 4.2.1. Test of Level-1 Hypotheses

**Figure 6:**
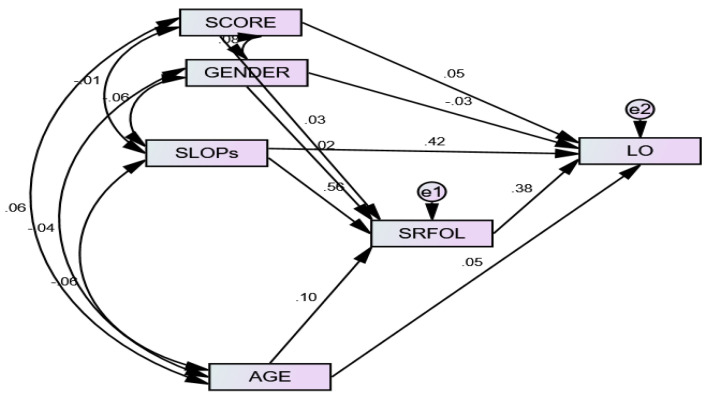
The structural model.

We tested the level-1 hypotheses in AMOS by specifying the direct and indirect effects of SOLPs on LO via SRFOL (Figure 6).

We also included control variables, such as the student’s gender, age, and overall score. We used Gaskin Estimand (Gaskination’s StatWiki, Provo, UT, USA) with 5000 bootstrapped samples and 95% bias-corrected confidence intervals for obtaining the indirect effect. The results showed that among three control variables, students’ age has significantly and positively affected their readiness for online learning (β = 0.10, *p* < 0.05). The students’ overall score has significantly and positively affected the LO (β = 0.05, *p* < 0.05). Besides these two significant effects, the remaining effects of controls were nonsignificant.

Table 5 results showed that SOLPs significantly and positively affected the LO (β = 0.42, *p* < 0.01) and SRFOL (β = 0.56, *p* < 0.01). SRFOL significantly and positively affected the LO (β = 0.38, *p* < 0.01). The indirect effect of SOLPs on LO via SRFOL was found significant (β = 0.33, *p* < 0.01). The follow-up analysis showed a partial mediating effect. Overall, the results supported the Level-1 hypotheses. The fit indices of the structural model presented in Table 2 showed a good fit.

#### 4.2.2. Test of Level-2 Hypothesis

The TOTR was conceptualized as a level-2 variable. We tested the level-2 hypothesis in HLM (Table 6). The interaction term of SOLPs and TOTR was computed by multiplying their scores. In HLM, the effects of SOLPs, TOTR, and interaction terms were specified, and the results were obtained [43]. The results show that SOLPs significantly and positively affected the LO (γ = 0.99, *p* < 0.01). The results also showed that the interaction term of SOLPs and TOTR significantly and positively affected the LO (γ = 0.15, *p* < 0.01). The results supported the moderating effect hypothesis (H5).

**Figure 7:**
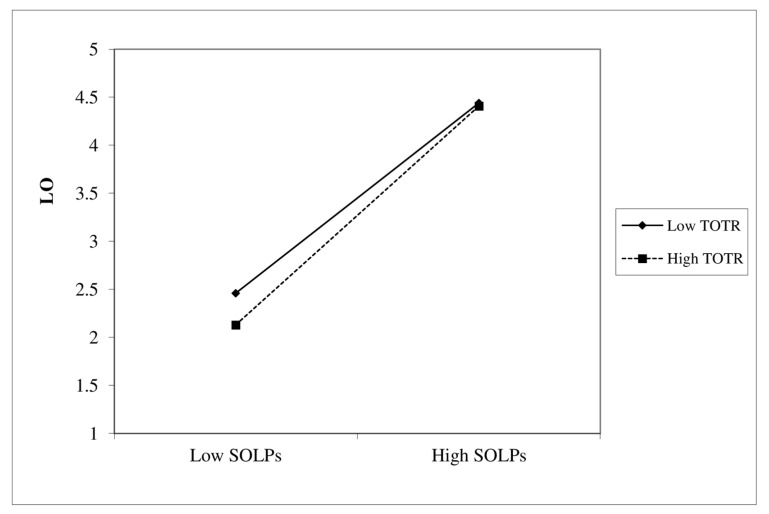
The moderating effect of TOTR on SOLPs and LO.

We conducted the slop analysis by plotting the combination of high and low degrees of SOLPs and TOTR on LO (Figure 7). We define high degree as one standard deviation above the mean (µ + 1σ) and low degree as one standard deviation below the mean (µ − 1σ). The graph shows that the LO is also the highest level at high degrees of SOLPs and TOTR and vice versa.

## 5. Discussion

Given the importance of online learning during COVID-19, particularly for the medical discipline [23], we examined the direct and indirect effects of medical SOLPs on LO via their readiness for online learning. We further hypothesized the moderating effect of TOTR on the relationship between SOLPs and LO. To test the hypotheses, we used a multilevel research design. The results supported the hypothesized relationships. Our study findings are consistent with theoretical assertions [13,16,19,27] and findings of earlier studies [13]. Particularly, our results showed that intrinsic motivation compared to extrinsic motivation strongly influences students’ attitudes to adopt an online learning environment to achieve LO. Our findings are consistent with Gupta’s [23] findings based on the Indian context. The findings of this research make meaningful contributions to theory and practice–given the importance of information technology for education [27,48], and medical education in particular [4,5].

### 5.1. Theoretical Contributions

Our study departs from the extant literature because of its focus on technology, especially for medical education. First, by unfolding the relationship between medical SOLPs and LO, this study lays the foundations for designing and implementing an online learning system that caters to the needs of medical discipline by focusing on both theory and practical aspects. Given the importance of intrinsic motivation to adopt an online learning environment, the synchronous teaching mode can be supplemented with an asynchronous mode such as providing technological-based and self-paced learning resources to the students, such as surgical videos, telehealth, telemedicine, and online practice questions could develop positive perceptions of medical students towards online learning and help them achieve LO [7]. The medical colleges should introduce and implement innovative learning opportunities for the students, such as community work services, remote elective coursework and creating patient education material.

Second, this research contributes to the limited literature on medical SRFOL [49] especially in a low-tech country context such as Pakistan. Our findings showed SOLPs are positively associated with online learning readiness, which enhanced the LO [13,27,49]. Consistent with the self-determination theory [16], our results imply that medical SOLPs increase their intrinsic motivation to undertake online learning activities to achieve LO. Such that when students perceive they can manage online learning, they become ready to engage in online learning that helps in the achievement of LO.

Finally, this research establishes TOTR as a boundary condition on the direct relationship between medical SOLPs and LO. In line with the self-determination theory [16] and constructivist theory [19], we examined the joint effect of students’ intrinsic motivation (SOLPs) and extrinsic motivation (TOTR) on LO. The findings imply that the students with positive perceptions of online learning achieve LO when TOTR are high. Our research contributes to the limited but growing research on TOTR during COVID-19 [14] and contributes to the general literature on technology readiness [2,35].

### 5.2. Practical Implications

Our study’s findings have important implications for educators and policymakers to design and implement an online learning system in medical institutions. Online learning perceptions encompass five factors: adaptability, accessibility, interactivity, knowledge acquisition, and ease of loading, which enable the students to achieve LO. Therefore, these factors are the prerequisite for an online learning system to build students’ confidence in online learning and academic achievements. Thus, faculty members and medical colleges administrators should consider these factors for designing and implementing an online learning system. The medical colleges should arrange clinical placements of their students in hospitals and community healthcare systems that are near to students’ home locations to supplement theory with practice.

This research found that online learning readiness significantly contributes to achieving the LO. Online learning for medical students accompanies many challenges, such as poor motivation, difficulty in understanding the content, limited focus on practical orientation, and lack of technology skills [50]. In a developing country like Pakistan, poor connectivity and a lack of technical support at home are the major barriers to online education. Therefore, educators need to develop online learning modules based on students’ ease, accessibility, and interactivity to enhance SRFOL, which enables them to achieve LO. In addition, TOTR (technical competence, time management, course design, and course communication) positively moderates the relationship between SOLPs and LO. Thus, administrators should also encourage and reward the faculty members for developing online learning materials that help students in achieving the LO. They should arrange the training programs for the teachers and students to use the online learning platforms that maximize the LO in this challenging time.

### 5.3. Limitations and Future Research Directions

This research is not without limitations. First, we collected data from fourth- and fifth-year medical students in the Punjab province that could limit its generalizability. Sample should be taken from diverse disciplines such as pharmacy, engineering, sciences, computer science, and social sciences to enhance generalizability and determine between variability if that exists.

Second, our study did not specify and determine the students’ perceptions about the effectiveness of the online learning system that was in place. Therefore, we suggest undertaking in-depth studies to determine the students’ perceptions of the learning management system and its effectiveness.

Third, we use TOTR as the boundary condition of SOLPs and LO. We suggest exploring the other factors that influence phenomena under investigation. For example, computer literacy, peer readiness for online learning, and institutional support are important factors that could moderate the relationship between online learning perceptions and LO.

Fourth, our results showed that, for our sampled group, intrinsic motivation such as SOLPs had a strong effect on LO as compared to extrinsic motivation (TOTR). The HLM results showed a nonsignificant effect of TOTR on LO. Therefore, it requires further validation for disciplines other than medical and contexts, such as face-to-face and different cultural settings.

Finally, our study considered the positive aspects of online learning, such as adaptability, accessibility, interactivity, knowledge acquisition, and ease of loading to determine the SRFOL. How the negative psychological factors such as stress, fear, and uncertainty influence the students’ perceptions of online learning during COVID-19, is a promising area of future inquiry [51]. We invite future researchers to study their effects in determining SOLPs.

## 6. Conclusions

In this time-lagged and multilevel research, we determined the direct and indirect effects of medical SOLPs on LO via SRFOL. We also introduced TOTR as a boundary condition of SOLPs and LO. The results showed support for our study’s hypotheses. The results imply that students with positive perceptions of online learning were ready to engage in online learning to achieve LO. The TOTR also augmented the effects of SOLPs on LO. In the end, we presented the implications of our study’s findings and suggested promising areas for future inquiry. In concluding remarks, our study persuades the medical institutions’ administrators and teachers to plan and implement a user-friendly online learning system that fulfills the medical education requirements in terms of both theory and practice to prepare healthcare professionals to meet the urgent needs of the healthcare system during COVID-19.

## Figures and Tables

**Table 1:** Second-order measurement model results (Level-1).

Latent Construct	Dimensions/Items	Factor Loading	AVE	CR	Cronbach’s Alpha (α)
Students’ online learning perceptions (SOLPs)	Accessibility	0.74			
	Interactivity	0.70			
	Adaptability	0.73			
	Knowledge acquisition	0.76			
	Ease of loading	0.72	0.54	0.85	0.83
Students’ readiness for online learning (SRFOL)	Computer & internet self-efficacy	0.80			
	Self-directed learning	0.65			
	Learner’s control	0.70			
	Motivation for learning	0.72			
	Online communication self-efficacy	0.67	0.51	0.84	0.79
Learning outcomes (LO)	LO1	0.83			
	LO2	0.84			
	LO3	0.84			
	LO4	0.89			
	LO5	0.86			
	LO6	0.86	0.73	0.94	0.94

AVE stands for average variance extracted score, and CR stands for composite reliability.

**Table 2:** Model Fit indices.

Fit Indices	Eleven-Factor Model (Level-1)	Three-Factor Model (Second-Order Level-1)	Four-Factor Model (Level-2)	Single-Factor Model (Second-Order Level-2)	Structural Model
CMIN/DF	1.91	1.98	1.36	1.35	1.21
RMR	0.02	0.03	0.05	0.05	0.02
RMSEA	0.04	0.04	0.06	0.06	0.02
IFI	0.95	0.94	0.94	0.94	0.97
CFI	0.94	0.94	0.93	0.93	0.95
TLI	0.95	0.94	0.94	0.94	0.97

CMIN/DF stands for minimum discrepancy per degree of freedom, RMR stands for root mean residual, RMSEA stands for root-mean-square error of approximation, IFI stands for incremental fit index, CFI stands for comparative fit index, and TLI stands for Tucker-Lewis’s index.

**Table 3:** Second-order measurement model results (Level-2).

Latent Construct	Dimensions	Factor Loading	AVE	CR	Cronbach’s Alpha (α)
Teacher’s online teaching readiness (TOTR)	Course design	0.81			
	Course communication	0.77			
	Time Management	0.50			
	Technical competence	0.75	0.51	0.80	0.79

Confirmatory factor analysis supported the factor structure of second-order latent constructs (e.g., SOLPs, SRFOL, and TOTR) to compute the composite score of these variables for further analysis. The comparisons of the squared roots of AVE scores with paired correlation coefficients showed that the squared roots of AVE scores were greater than the paired correlation coefficient that supported the discriminant validity (Table 4).

**Table 4:** Descriptive statistics, correlation, and the square root of average variance extracted scores.

Variables	Mean	SD	1	2	3	4	5	6	7
Student’s gender	-	-	-						
Student’ age	25.41	1.97	−0.04	-					
Student’s overall score	68.63	4.88	0.08	0.06	-				
SOLPs	3.37	0.53	−0.06	−0.06	−0.01	(0.73)			
SRFOL	3.36	0.45	−0.02	0.07	0.03	0.55 **	(0.71)		
TOTR	3.92	0.56	−0.03	0.09 *	0.09 *	0.26 **	0.24 **	(0.72)	
LO	3.45	0.84	−0.06	0.06	0.06	0.62 **	0.61 **	0.32 **	(0.85)

** *p* < 0.01. * *p* < 0.05. The values in the diagonals are the squared root of average variance extracted scores. Mean is the arithmetic mean. SD stands for standard deviation. 1-student’s gender, 2-student’s age, 3-student’s overall score, 4-SOLPs,5-SRFOL, 6-TOTR, 7-LO.

**Table 5:** Structural model results for direct and indirect effects.

Paths Tested	SRFOL			LO		
	β	Lower Bound	Upper Bound	β	Lower Bound	Upper Bound
Controls						
Gender	0.02	−0.06	0.09	−0.03	−0.09	0.03
Age	0.10 *	0.03	0.17	0.05	−0.01	0.11
Overall score	0.03	−0.04	0.10	0.05 *	0	0.11
Direct Effects						
SOLPs	0.56 **	0.47	0.63	0.42 **	0.33	0.49
SRFOL				0.38 **	0.30	0.46
Indirect Effects						
SOLPs --> SRFOL --> LO				0.33 **	0.25	0.44

** *p* < 0.01. * *p* < 0.05.

**Table 6:** HML results for moderating effects of TOTR (Level-2) on SOLPs and LO (Level-1).

Predictors	LO (γ)
Intercept	3.45 (0.03) **
Predictor at Level-1	
SOLPs	0.99 (0.06) **
Predictors at Level-2	
TOTR	−0.18 (0.10)
SOLPs × TOTR	0.15 (0.02) **
R^2^	0.38
χ^2^	90.92 **

Notes: Standard errors are reported in parentheses; R^2^ is calculated using Kreft and de Leeuw [47]. ** *p* < 0.01.

## Data Availability

The data of current study can be obtained from corresponding author.

## Appendix A

**Table A1:** Measurement Scales.

Independent Variable: Students’ Online Learning Perceptions: Wei and Chou [13]	
Accessibility (SAC)	AC1. Online learning provides various multimedia learning resources.
	AC2. Online learning provides various online resources.
	AC3. Online learning enables me to retrieve and obtain more learning resources.
	AC4. Online learning enables me to share and exchange resources.
Interactivity (SINT)	INT1. Online learning enables me to interact directly with other learners.
	INT2. Online learning can encourage interaction between instructors and students.
	INT3. Online learning can shorten the distance between instructors and students.
	INT4. Online learning enables me to meet more classmates or peers with the same interests or habits.
	INT5. Online learning provides sufficient discussion opportunities.
	INT6. Online learning provides convenient tools to communicate with other learners.
Adaptability (SAD)	ADA1. Online learning enables me to decide on the best time to learn.
	ADA2. Online learning enables me to decide on the best location to learn.
	ADA3. Online learning enables me to repeatedly review learning materials.
	ADA4. Online learning overcomes time and place constraints.
Knowledge Acquisition (SKA)	KA1. Online learning can broaden my common knowledge base.
	KA2. Online learning enables me to learn more about the knowledge that I desire to learn.
	KA3. Online learning can expand my academic knowledge capacity.
	KA4. Online learning is an effective learning style.
	KA5. Online learning enables an abstract idea or concept to be presented in a concrete manner.
Ease of Loading (SEL)	EL1. Online learning environments lead to less pressure to catch up with a course schedule.
	EL2. Online learning environments are less stressful.
	EL3. Online learning environments place less pressure on exams and assessments.
	EL4. Online learning environments can effectively reduce learning burden.
**Mediator Variable: Students’ Readiness for Online Learning: Hung et al. [27]**	
Computer/Internet Self-efficacy (SSE)	SCE1. I feel confident in performing the basic functions of Microsoft Office programs (MS Word, MS Excel, and MS PowerPoint).
	SCE2. I feel confident in my knowledge and skills of how to manage software for online learning.
	SCE3. I feel confident in using the Internet (Google, Yahoo) to find or gather information for online learning.
Self-directed learning (SSL)	SDL1. I carry out my own study plan.
	SDL2. I seek assistance when facing learning problems.
	SDL3. I manage time well.
	SDL4. I set up my learning goals.
	SDL5. I have higher expectations for my learning performance.
Learner’s control (SLC)	LC1. I can direct my own learning progress.
	LC2. I am not distracted by other online activities when learning online (instant messages, Internet surfing).
	LC3. I repeat the online instructional materials on the basis of my needs.
Motivation for learning (SLM)	ML1. I am open to new ideas.
	ML2. I have motivation to learn.
	ML3. I improve from my mistakes.
	ML4. I like to share my ideas with others.
Online communication self-efficacy (SSCE)	OCE1. I feel confident in using online tools (email, discussion) to effectively communicate with others.
	OCE2. I feel confident in expressing myself (emotions and humor) through text.
	OCE3. I feel confident in posting questions in online discussions.
**Dependent Variable: Learning Outcomes (SLO); Source: Chiu et al. [42]**	
	LO1. I gain medical knowledge that I did not know before.
	LO2. I learn how to apply my knowledge to solve different medical problems which happen in a real-life.
	LO3. I learn how to practice and apply the correct approach to solving medical problems.
	LO4. I learn how to study systematically, such as using a concept map.
	LO5. I learn how to communicate with patients and their family members, such as explaining medical conditions to them.
	LO6. I learn how to cooperate with other medical professionals to solve problems.
**Moderator: Teacher Readiness to Teach Online; Source: Martin et al. [36]**	
Course Design (FCD)	CD1. Create an online course orientation (e.g., introduction, getting started).
	CD2. Write measurable learning objectives.
	CD3. Design learning activities that provide students opportunities for interaction (e.g., discussion forums, wikis).
	CD4. Organize instructional materials into modules or units Create instructional videos (e.g., lecture video, demonstrations, video tutorials).
	CD5. Use different teaching methods in the online environment (e.g., brainstorming, collaborative activities, discussions, presentations).
	CD6. Create online quizzes and tests.
	CD7. Create online assignments.
	CD8. Manage grades online.
Course Communication (FCC)	CC1. Send announcements/email reminders to course participants.
	CC2. Create and moderate discussion forums.
	CC3. Use email to communicate with the learners.
	CC4. Respond to student questions promptly (e.g., 24 to 48 h).
	CC5. Provide feedback on assignments (e.g., 7 days from submission).
	CC6. Use synchronous web-conferencing tools (e.g., Adobe Connect, Webex, Blackboard Collaborate, Skype, Zoom, Google meet).
	CC7. Communicate expectations about student behavior (e.g., netiquette).
	CC8. Communicate compliance regarding academic integrity policies.
	CC9. Apply copyright law and fair use guidelines when using copyrighted materials.
	CC10. Apply accessibility policies to accommodate student needs.
Time Management (FTM)	TM1. Schedule time to design the course prior to delivery (e.g., a semester before delivery).
	TM2. Schedule weekly hours to facilitate the online course.
	TM3. Use features in a learning management system in order to manage time (e.g., online grading, rubrics, SpeedGrader, calendar).
	TM4. Use facilitation strategies to manage time spent on course (e.g., discussion board moderators, collective feedback, grading scales).
	TM5. Spend weekly hours to grade assignments.
	TM6. Allocate time to learn about new strategies or tools.
Technical Competence (FTC)	TC1. Complete basic computer operations (e.g., creating and editing documents, managing files and folders).
	TC2. Navigate within the course in the learning management system (e.g., Moodle, Canvas, Blackboard, etc.).
	TC3. Use course roster in the learning management system to set up teams/groups.
	TC4. Use online collaborative tools (e.g., Google Drive, Dropbox).
	TC5. Create and edit videos (e.g., iMovie, Movie Maker, Kaltura).
	TC6. Share open educational resources (e.g., learning websites, Web resources, games and simulations).
	TC7. Access online help desk/resources for assistance.
